# The value of postpartum ultrasound for the diagnosis of retained products of conception: A systematic review

**Published:** 2017-12

**Authors:** J De Winter, H De Raedemaecker, J Muys, Y Jacquemyn

**Affiliations:** Antwerp University Hospital UZA, Department of Obstetrics and Gynaecology, Wilrijkstraat 10, 2650 Edegem,Belgium

**Keywords:** EEC, endometrial echo complex, postpartum, retained placenta, retained products, ultrasound

## Abstract

**Background:**

The goal of this review is to evaluate the value of ultrasound for detection of retained products of conception (RCOP) after delivery.

**Methods:**

A systematic search was performed using ‘postpartum’, ‘retained placenta’, ‘retained products’ and ‘ultrasound’ resulting 82 publications, after screening titles and abstracts, 30 remained.

**Results:**

On gray scale ultrasound, one must be focus on a thickened endometrial echo complex (EEC) with a cut off value of 10 mm and on an intracavitary mass. If these features are not visible, RPOC is rare. However, these findings are neither specific nor conclusive for RPOC and can even be seen in a normal postpartum uterus. Detection of hypervascularity in a thickened EEC or intracavitary mass with color Doppler ultrasound is very sensitive for RPOC but still not specific nor can it exclude RPOC. MRI seems best in differentiating RPOC, arteriovenous malformations and gestational trophoblastic disease.

**Conclusion:**

There is no consensus on a standardised method for postpartum ultrasound. More research and standardization are necessary to differentiate of normal and pathological findings in the postpartum uterus.

## Introduction

Retained products of conception (RPOC) complicate around 1% of all deliveries (Weissbach et al., 2015) and are defined as remnants of placental trophoblastic origin (Laifer-Narin et al., 2014). If undiagnosed and not treated properly they can result in a secondary postpartum haemorrhage as a result of subinvolution, infections and intrauterine adhesions (Weissbach et al., 2015). Postpartum haemorrhage is the leading cause of maternal morbidity and mortality worldwide (Urner et al., 2014). Therefore, it is important to recognise signs and symptoms timely, which could be caused by retained products in a woman with postpartum complaints. There are variable presentations of RPOC but the most common clinical symptoms are an enlarged uterus, an open cervical os (Neill et al., 2002), abdominal pain, vaginal bleeding and fever (Adkins et al., 2016). Unfortunately, these symptoms are very unspecific, which makes the diagnosis even harder. There are multiple diseases causing them, and even in a normal postpartum uterus they can occur (Durfee et al., 2005). This brings us to the fact that knowledge of the normal postpartum period is indispensable in diagnosing a pathological condition. More and more, ultrasound seems to be a useful tool for diagnosing RPOC and it is assumed to be more accurate than a clinical presentation alone (Sellmyer et al, 2013). The difficulty with ultrasonic findings though is that they are based on signs that can also be found in the normal postpartum uterus. Different studies also show different findings as most sensitive for RPOC, which makes it all even more confusing.

Known risk factors for RPOC are nulliparity, maternal age, induction of labour, uterine surgery in the woman’s history (Weissbach et al, 2015) and placenta accreta (Guarino et al., 2015). The golden standard in the treatment of RPOC is dilatation and curettage (D&C) (Scribner and Fraser, 2016). However, this intervention is not without risks. Further complications are seen in 7% and include perforation of the uterus, cervical laceration and subsequent synechia formation (Durfee et al., 2005) sometimes even leading to a hysterectomy (Mulic-Lutvica and Axelsson, 2006). In order to avoid unnecessary interventions and possible complications, we need to be as sure as possible about the diagnosis of RPOC before considering this treatment. Otherwise a more conservative approach should be maintained.

## Methods

Between September and October 2016 we searched PubMed using the following Mesh Terms: retained products, retained placenta, postpartum, and ultrasound. We did not limit the search in time but we narrowed it down to English articles. Our inclusion criteria were women with a gestational age of more than 24 weeks, who delivered vaginally or with a caesarean section. Exclusion criteria were miscarriage and stillbirth. We also used OVID and Web Of Science as other databases but no new relevant articles were found.

**Figure 1:**
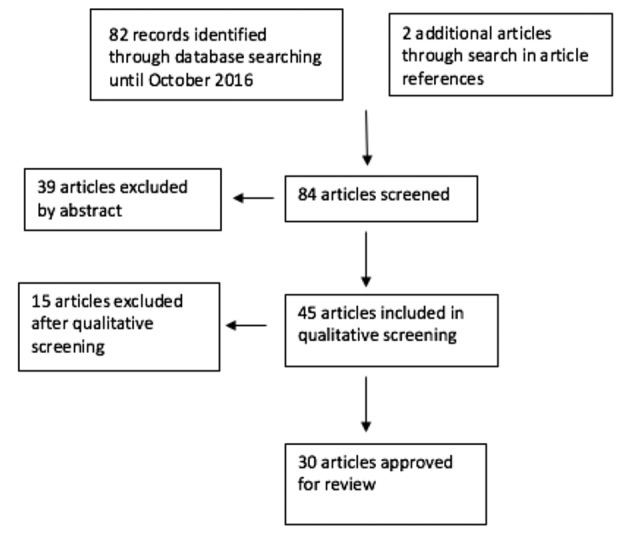
— Flowchart of Study Selection.

Next, we screened the articles for relevance based on their title and abstract and we excluded 39 articles, which left us with 45 articles to be included in our literature study. We then evaluated these articles with a self-drawn up checklist based on the evaluation forms from the Dutch Cochrane and excluded 15 more. Eventually, 30 articles remained useful for our literature review. Figure 1 shows a flow chart of the study selection.

## Results

### The normal postpartum uterus

In order to be able to differentiate a pathological situation from a normal one, we must understand what to expect in an uncomplicated postpartum period. The postpartum period can be defined as a period of 6 - 8 weeks following delivery during which the uterus undergoes involution and returns to its original state and the endometrium resumes to its normal thickness. (Mulic-Lutvica et al., 2001; Steinkeler et al., 2012). A wide variability of normal postpartum ultrasonic findings is presented in the review of Steinkeler et al. (2012) Simple fluid, echogenic material consistent with blood products and avascular echogenic material could be normal and should not be concluded as RPOC per se. A correlation with the patient’s history and clinical symptoms should always be performed.

Sokol et al. (2004) performed a prospective observational study on the normal sonographic findings after uncomplicated vaginal delivery. They measured endometrial stripe thickness, maximum uterine length and maximum uterine width in the sagittal plane with trans-abdominal ultrasound in 40 women within 48h after delivery. Uterine content and uterine size were estimated based on calculations. Patients were asked to quantify their bleeding with the number of used pads and keep a diary for 6 weeks. In this group postpartum bleeding became less at a mean of 7.8 ±7.0 days with a mean duration of 25.6 ±10.6 days. Three patients developed a fever but this was caused by a mastitis rather than being a normal postpartum symptom. Results for the sagittal measurements were as followed: mean endometrial stripe thickness was 1.1 ±0.6 cm; mean uterine length 16.1 ±1.7 cm; mean uterine width 8.1 ±1.0 cm and mean uterine size 140.6 ± 27.7 cm2. In 40% of the women there was echogenic material in the endometrial cavity noted with a mean size of 12.7 ± 6.9 cm2. No association with heavier or prolonged bleeding was found. They did find out that patients with more than 10 days of heavy bleeding (> 4 pads per day) had thicker endometrial stripes but there was no correlation with the duration of their bleeding. None of the women needed medical care for their bleedings. These findings suggest that a thickened endometrial stripe and echogenic material in the endometrial cavity within 48h postpartum can be normal findings. They can be explained by blood clots and will therefore not need any further interventions.

Edwards et al. (Edwards and Ellwood, 2000) studied the trans-abdominal appearances of the postpartum uterus later on in the uncomplicated postpartum period. They scanned 40 women at day 7, 14 and 21 after a normal vaginal delivery. They estimated uterine size and content and documented appearances in the uterine cavity. The women also kept a diary for 6 weeks describing the amount of vaginal bleeding classified as light, medium or heavy. They found that the mean duration of postpartum bleeding was 24.5 days, which is in agreement with what Sokol et al. (2004) measured. They also found echogenic masses in the uterine cavity: 51% at 7 days postpartum, 21% at 14 days postpartum and 6% at 21 days postpartum. Here too, was no significant association found between bleeding duration and an echogenic mass on the postpartum ultrasound, which raises questions on the importance of finding an echogenic mass in the diagnosis of RPOC. Even more, there was no statistical correlation between duration or quantity of bleeding and the uterine or cavity volume at any of the three examinations.

One year later, a study was published (Mulic- Lutvica et al., 2001) where the main focus was the anteroposterior diameter of the uterus and the uterus cavity. Six ultrasound scans took place at day 1, 3, 7, 14, 28 and 56 postpartum. The first four scans were performed trans-abdominally, the second two trans- vaginally. The maximum anteroposterior diameter of the uterus started at 92.0 mm and progressively diminished to 38.9 mm on day 56. The maximum anteroposterior diameter of the uterus cavity went from 15.8 mm on day 1 to 4.0 mm on day 56. On Day 1, 92,9% of the women had an empty cavity, but this number decreased with time and at day 7 only 9,8% of them had an empty cavity. From then on, the number increases again and at day 56, 95,1% of the women had an empty cavity again. They concluded it was difficult to generate a standardized protocol on how the postpartum uterus should look like on ultrasound.

### Retained products of conception

“Retained products of conception” is any intrauterine tissue, which developed during pregnancy and persisted after delivery. When chorionic villi are found post partum, placental tissue must be present (Sellmyer et al., 2013). It is seen in about 1% of all deliveries (Laifer-Narin et al., 2014; Weissbach et al., 2015) and the incidence varies with gestational age; it occurs most frequent after second trimester delivery or termination of pregnancy (Sellmyer et al., 2013). Patients often present with abdominal pain, fever and postpartum haemorrhages (PPH) (Adkins et al., 2016). PPH can either be primary or secondary; any blood loss greater than 500mL in the first 24h postpartum is called a primary haemorrhage. Disproportionate blood loss after 24h until 6 weeks postpartum is categorised as a secondary haemorrhage. Both types of PPH are most frequently caused by RPOC (Sellmyer et al., 2013). Because of RPOC the uterus cannot involute and the bleeding continues, making a uterine curettage indispensable (Shaamash et al., 2007).

Several researchers describe multiple risk factors for RPOC including failure to progress during delivery, instrumental delivery and placenta accreta (Guarino et al., 2015; Sellmyer et al., 2013). In placenta accreta there is an abnormally invasive placental implantation in the decidua basalis. An important known risk factor is a previous uterine scar, mainly caused by caesarean deliveries. Given the rise in caesarean sections, there has been a 10-fold increase over the last 50 years of the incidence of placenta accreta (Guarino et al., 2015). Nevertheless, there does not seem to be a direct significant association between retained products and a vaginal or caesarean delivery (Pather et al., 2005).

Other risk factors of RPOC are an older maternal age, labour induction and nulliparity (Weissbach et al., 2015). Although others say that multigravida is more of a risk factor since placenta accreta is more common in multigravida and a multigravida uterus is less able to contract as well (Van den Bosch et al., 2002). Necessity of a blood transfusion in the early postpartum also seems to be an indication for possible retained products (Van den Bosch et al., 2002). This could however be explained by the postpartum haemorrhages frequently seen with RPOC.

### Ultrasound findings of RPOC

When a patient presents with PPH and a high risk for RPOC, it is important to perform a first investigation. Sellmyer et al. (2013) conclude that ultrasound can be a very useful tool in this process and it seems to be more accurate than clinical symptoms alone for the diagnosis. They found a thickened endometrial echo complex (EEC) to be the most sensitive (sensitivity 80%) ultrasound finding for RPOC, with a cut-off value of 10 mm. This is in agreement with what Pather et al. (2005) found, where a thickened endometrium (cut-off range 10-40 mm) was said to be the most accurate individual predictor of RPOC (positive predictive value 67%). A higher positive predicted value can be obtained if there is also an echogenic focus found (positive predictive value 80%). The specificity of this finding on the other hand is very low (specificity 20%) since a thickened EEC can also be seen in postpartum patients without RPOC. They state that color Doppler is essential for further clarifying the diagnosis. A second, very sensitive finding (sensitivity 79%) according to them, is an endometrial mass. They do admit that different sensitivities are measured in various studies but they explain this by the different definitions the examiner can give to an endometrial mass. Kamaya et al. (2009) conclude as well that RPOC can best be diagnosed with images obtained using gray-scale ultrasound and color Doppler. Attention should be paid to endometrial thickness, focal masses and the degree of vascularisation within the endometrial cavity. There is a poor consensus for the cut-off value of the endometrial thickness found in literature; the range varies from 8 mm (34% false positive rate) to 13 mm (85% sensitivity, 64% specificity). A proposed threshold is 10 mm, as stated before.

Matijevic et al. (2009) did a prospective cohort study on the accuracy of sonographic and clinical parameters in the prediction of RPOC. Ninety-three women in their late postpartum period (usually 2-3 days postpartum) who were suspected for RPOC, based on clinical or sonographic predictors, underwent evacuation of their uterus followed with histopathological examination. They defined clinical predictors for RPOC as vaginal bleeding, lower abdominal pain, fever (>38°C) and cervical dilatation. Sonographic predictors were the following: endometrial mass with hyperechoic, hypoechoic or mixed echogenic patterns in the uterine cavity greater than 10 mm. Histopathologic examination confirmed RPOC in 58% of the cases. The relationship between histologically confirmed RPOC and clinical and sonographic parameters was studied and sonography turned out to have the highest sensitivity (98,1%) with an endometrial mass as the most sensitive finding. When no endometrial mass can be found and the endometrium thickness is less than 10 mm, RPOC are extremely rare. Abdominal pain had the lowest sensitivity (7,4%) but also the highest specificity (79,5%). Sonography had a 30% higher sensitivity rate than cervical dilatation but the specificity was similar. Clinical signs and symptoms alone did not appear to be statistically significant predictors of RPOC. Also, PPH turned out to be the most important indication for hospital referral and RPOC was histologically confirmed in 45% of the cases. Fever and abdominal pain seem to be less likely related to RPOC then bleeding, and the combination of these three symptoms decreases chances of RPOC because this combination is more suggestive for endometritis.

Mulic-Lutvica and Axelsson (2006) performed a prospective observational study of 79 women with secondary postpartum haemorrhages. The women were examined by ultrasound on the day they presented with symptoms and on postpartum day 1, 3, 7, 14, 28 and 56. They were divided into two groups according to the treatment of choice. Group one (22,8%) underwent a surgical evacuation, group two a conservative treatment (77,2%). In group one, uterine sizes tended to be larger and 94,4% of the women had a well-circumscribed, lobulated echogenic mass in their uterus. 82,3% of them had histologically proven retained products of conception. And none of the women without an echogenic mass, turned out to have RPOC. So, in accordance with what Matijevic et al. (2009) found, they too suggested that an echogenic mass is strongly associated with RPOC. The same conclusion can be made out of Shen et al. (2003) results. They performed postpartum ultrasounds on 39 women who were suspected for RPOC as examination of the placenta showed incomplete placentas. Twenty-one women showed an intrauterine mass with a thickness of > 10 mm on ultrasound, and in 15 of them RPOC was histopathologically confirmed. This makes that the sensitivity of ultrasound for predicting RPOC is 93,8% with a specificity of 73,9%. Neill et al. (2002) found that echogenic foci on ultrasound have approximately the same sensitivity (93%) and specificity (62%). Nevertheless, they do point out that the positive predictive value of ultrasound assessment is pretty poor (PPV 46%). A negative scan however is reassuring (NPV 96%). Their conclusion is that combining ultrasound and clinical assessment improves diagnostic accuracy when they are both positive or negative, but accuracy is limited when there is a disagreement between clinical and ultrasound findings.

Another study (Hoveyda and MacKenzie, 2001) compared histological outcomes in women admitted for PPH, either with or without a previous ultrasound scan. Seventy-five women underwent a uterine evacuation, 46 of them were referred after a positive ultrasound for RPOC, and the rest was diagnosed clinically. Amongst the women without pre-operative scan, 33% received a confirmation of RPOC compared with 37% in the women who were send in after a scan. It is clear that ultrasound did not provide a significant advantage over clinical assessment for RPOC. Researchers Hertsberg and Bowie (1991) were the first to classify postpartum ultrasound scans in categories. Five categories were identified:

1. Normal uterine stripe with a thickness of the uterine cavity less than 15 mm
2. Endometrial fluid
3. Echogenic mass enlarging the uterine cavity (AP diameter 15 mm or greater)
4. Hyperechoic foci/no mass (AP diameter less than 15 mm)
5. Heterogeneous mass composing of a mixed pattern of echogenic and echogenic areas enlarging the endometrial cavity (15 mm or greater).

Fifty-three patients were referred for ultrasound evaluation for RPOC. There is a strong correlation found between an echogenic mass found on ultrasound and pathologically proven RPOC. In 8 of these 10 patients the mass had a stippled appearance due to hyperechogenic foci. These foci were also regularly seen in patients without RPOC who had undergone recent uterine instrumentation. To avoid confusion, it is therefore better to perform the ultrasound before instrumentation since this can introduce air resembling the hyperechoic foci seen with RPOC. Patients with pathologically proven RPOC also appeared to have significantly greater AP diameters of the uterine cavity (21,4 mm) compared to those without RPOC (8,3 mm).

A few years later Carlan et al. (1997) performed a similar study. They performed postpartum ultrasounds on 127 women, within 5 minutes after placental delivery, followed by a manual exploration and sponge curettage. Then, they let an investigator without knowledge of the pathologic results categorize the scans into 4 categories resembling these of Hertzberg and Bowie (1991):

1. A normal uterine “stripe”: a hypo- or hyperechoic linear region over the entire length of the uterus. The thickness of the stripe needs to be less then 15mm.
2. Endometrial fluid: a fluid density space in the uterus of any size
3. An echogenic mass: calcifications inside a solid mass, stretching the endometrial cavity 15 mm or more
4. A heterogeneous mass: endometrial cavity was enlarged > 15 mm by heterogeneous material of mixed densities.

They considered scans in category 3 and 4 as positive for the diagnosis of RPOC. Twenty-four (18%) patients had histologically confirmed RPOC. They were previously classified in the following categories: a normal uterine stripe in 37,5%, only fluid in 16,6%, an echogenic mass in 25% and a heterogeneous mass in 21%. The sensitivity, specificity, positive and negative predictive value of ultrasound detection for RPOC, using these categories are respectively: 44%, 92%, 58% and 87%. We notice a discrepancy in sensitivity and specificity comparing to previous discussed studies (Neill et al., 2002; Shen et al., 2003), this could however be explained by a different time period used to perform the ultrasounds. Important is also that a normal uterine stripe does not exclude RPOC as 37,5% of histologically proven retained products appeared as a normal stripe. They conclude that their results do not support the high expectations of ultrasound in diagnosing RPOC, but they do admit there is a distinct correlation between an echogenic mass and RPOC as proved by other studies.

A ‘Summary of selected studies for the diagnosis of RPOC’ is shown in Table I and ‘Findings from selected studies in the diagnosis of RPOC’ are shown in Table II.

**Table I:** Summary of selected studies for the diagnosis of RPOC.

Study	Year	Study Size	Characteristics	Conclusion
Sokol et al	2004	40	Normal trans-abdominal ultrasonographic findings of the uterus within 48h after un- complicated vaginal delivery	A thickened endometrial stripe and echogenic material in the endometrial cavity within 48h postpartum can be normal findings.
Edwards & Ellwood	2000	40	Normal trans-abdominal ultrasonographic findings of the uterus in the postpartum period on day 7, 14 and 21 after uncompli- cated vaginal delivery	No significant association found between bleeding duration and an echogenic mass in the uterine cavity on the postpartum ultrasound
Mulic- Lutvica et al	2001	42	Normal ultrasonographic findings of the uterus in the postpartum period, trans-ab- dominal on day 1,3, 7 and 14 and trans-vag- inal on day 28 and 56, after uncomplicated vaginal delivery	A standardized protocol on how the postpartum uterus should look like is difficult to generate on ultrasound.
Sellmyer et al	2013		Physiologic, Histologic and Imaging fea- tures of retained products of conception	A thickened EEC (range 8-13mm) on gray-scale US is the most sensitive finding of RPOC (80%). This or an endometrial mass with vascularity on Doppler US allows confident diagnosis of RPOC.
Pather et al	2005	2000	A retrospective chart review of patients who underwent a postpartum curettage. They want to assess which features are best to predict the presence of RPOC.	Thickened EEC (range 10-40 mm) is the most accu- rate individual predictor of RPOC with a PPV of 67% and a PPV of 80% in combination with a echogenic focus. The specificity is low.
Kamaya et al	2009	269	Postpartum ultrasonographic images ob- tained with gray-scale US and color Dop- pler, with attention for masses, vascularity and EEC	RPOC can best be diagnosed with images obtained from gray-scale US and color Doppler. The proposed threshold for thickened EEC is 10 mm.
Matijevic et al	2009	93	Examination of the accuracy of sonographic and clinical parameters in the prediction of RPOC after histopathological examination on women who were suspected for RPOC.	When no endometrial mass can be found and the EEC is less than 10 mm, RPOC is extremely rare.
Mulic- Lutvica & Axelsson	2006	79	Ultrasonographic findings on women with secondary PPH on the day they presented with PPH and on day 1, 3, 7, 14, 28 and 56 postpartum.	An echogenic mass is strongly associated with RPOC
Shen et al	2003	39	Ultrasonographic findings on women sus- pected with RPOC after examination of the placenta showed incomplete placentas	Ultrasound for predicting PROC: sensitivity 93,8% and specificity 73,9%
Neill et al	2002	53	Comparison of the diagnostic accuracy of clinical assessment with transabdominal ultrasound in the management of secondary PPH expected to be caused by RPOC	Combining ultrasound and clinical assessement im- proves the diagnostic accuracy when they are both the same
Hoveyda & MacKenzie	2001	75	Comparison of histological outcomes in women with secondary PPH with or without an ultrasound scan	Ultrasound does not provide a significant advantage over clinical assessment of RPOC
Hertzberg & Bowie	1991	53	Classification of postpartum ultrasound scans in five categories	Strong correlation between echogenic mass with stippled appearance due to hyperechogenic foci on US and RPOC
Carlan et al	1997	127	Postpartum ultrasounds performed within 5 minutes after placental delivery, followed by a manual exploration and sponge curet- tage.	Normal uterine strip does not exclude RPOC. Correla- tion between echogenic mass and RPOC.

**Table II:** Findings from selected studies in the diagnosis of RPOC.

Diagnostic	Sensitivity	Specificity	PPV °	NPV °	LR+ °
Sellmyer et al, 2013	- Thickened EEC on US (cut off 10 mm)	80	20	...	<10 mm: 63-80	...
	- Endometrial mass on US	29-79	...	80	...	...
	- Vascularity on color Doppler	...	...	96	...	...
Pather et al, 2005	- Thickened EEC on US (cut off 10-40mm)	...	...	67	...	...
	- Combined with an echogenic focus	...	...	80	...	...
Kamaya et al, 2009	- Thickened EEC on US (cut off 8-13mm)	85	64	...	...	...
	- Echogenic mass on US	29	...	80	...	...
	- Vascularity on color Doppler	96	...	...	...	...
Matijevic et al, 2009	- PPH	77.7	30.8	74.2	25.8	1.12
	- lower abdominal pain	7.4	79.5	12.9	87.1	0.36
	- fever	27.7	64.1	31.1	68.8	0.77
	- cervical dilatation	70.3	30.7	69.8	30.1	1.01
	- sonographic character- istics	98.1	33.3	84.9	15.1	1.47
	- sonographic and color Doppler characteristics	66.6	69.2	51.6	48.3	2.16
Shen et al, 2003	- Echogenic mass on US (>10mm)	93.8	73.9	...	...	...
Neill et al, 2002	- Echogenic foci on US	93	62	46	96	...
Carlan et al, 1997	- Echogenic mass on US	44	92	50	87	...

° PPV: positive predictive value; NPV: negative predictive value; LR: likelihood ratio; EEC = endometrial echo complex; PPH = postpartum haemorrhages

### Other imaging techniques

Gray scale ultrasound is the most used imaging method in the diagnosis of retained placental tissue. On the ultrasound images you can see a thickened endometrial echo complex (EEC), ranging from 8 to 13 mm, or an intracavitary mass. However, there is no consensus about the correct diagnosis method for RPOC and in the literature there is said that gray scale US alone is not enough for a correct diagnosis of retained products of conception (Sellmyer et al., 2013). 3D ultrasound, color Doppler, CT, MRI and sonohysterography are other imaging techniques mentioned in the search for postpartum placental rest. Not all of them have the same value and not all of them are necessary to perform when looking for RPOC. In the following paragraphs we will discuss the different imaging techniques and their value.

Retained placental tissue has been suggested to be over-diagnosed, which leads to unnecessary invasive interventions (Belachew et al., 2015). Mostly, a two-dimensional gray scale ultrasound examination is performed, but Belachew et al. (2015) have looked into the value of three-dimensional ultrasound for a more accurate diagnosis of RPOC. They measured the volume of the uterine body and cavity using the VOCAL imaging program. Twenty-five women with postpartum haemorrhage were included in the study. All but one woman had a well-circumscribed echogenic mass often with lobulated appearance and calcifications and without fluid, viewed with 2D ultrasound. After this, the women were also examined with 3D ultrasound. They compared the volume of the uterine body and cavity from these women with reference values that were collected earlier. The women also underwent surgical resection of the mass and they performed a histological examination to confirm or exclude the presence of RPOC. They concluded that a large uterine cavity viewed with 3D ultrasound is an indication for RPOC. However, 3D ultrasound gives no -or little- extra diagnostic value or information compared to 2D ultrasound.

Different groups have researched the use of color Doppler ultrasound in the diagnosis of RPOC (Van den Bosch et al, 2002; Matijevic et al, 2009; Kamaya et al, 2009; Mulic-Lutvica et al, 2009; Steinkeler et al, 2012; Sellmyer et al. 2013; Urner et al., 2014).

With US color Doppler it is possible to see the vascularisation of the mass found with normal gray scale US, which increases the positive predictive value (PPV) for the diagnosis. If a vascularity is detected in a thickened EEC or intracavitary mass, the PPV increases to 96% (Sellmyer et al., 2013) and is highly suggestive for RPOC (Kamaya et al., 2009). Matijevic et al. (2009) found similar results. They published that the highest diagnostic accuracy for the detection of RPOC comes from the combination of gray scale US with color or pulsed Doppler US. If both these imaging techniques had positive findings, the LR+ increased with more than a factor 2 (Matijevic et al., 2009). Urner et al. (2014) talked in their systematic review about the research of Krapp et al. (), who examined patients in the third stage of labour with both a gray scale ultrasound and a color Doppler ultrasound. If there was a normal placental separation, the blood flow between the placenta and myometrium, demonstrated with color Doppler, stopped immediately after childbirth. In case of retained placenta, the blood flow from the myometrium deep into the placenta was persistent and hypervascularity was shown. The presence of Doppler flow in the outer and middle myometrium or over the whole thickness of the uterine wall was considered normal. The hypervascularity can be concentrated in a focal area or in a larger, transmural area. Both can be an indication of RPOC (Van den Bosch et al., 2002). The degree of vascularisation into the thickened EEC or mass is also important to be considered. This can be divided into different types:

- type 0 vascularity is defined as no vascularity and indicates no or avascular RPOC, which does normally not need an intervention and will resolve spontaneously;
- type 1 indicates a minimal vascularity with a PPV for RPOC greater than 90%;
- type 2 indicates a moderate vascularity and has a PPV of 100%;
- type 3 indicates a high vascularity also with a PPV of 100% (Sellmyer et al., 2013).

In type 0 there is no hypervascularity but this does not exclude RPOC, hypervascular areas are more commonly observed in patients with RPOC, but it is not a necessity. Steinkeler et al. agreed with the conclusion that the presence of an endometrial mass or hypervascularity seen on color Doppler can help the diagnosis of RPOC, but the absence of these features does not exclude RPOC. (Steinkeler et al., 2012) The conclusion of the research of Mulic-Lutvica et al. (2009) was that the use of color Doppler to observe hypervascular areas has limited powers in the diagnosis of RPOC. Also, Pather and colleagues (2005) found similar results and they even concluded that the PPV did not increase while using color Doppler. Finally, the fact that hypervascularity can be seen in many different diseases and in a normal postpartum placenta makes the diagnosis of RPOC with color Doppler more difficult.

Although ultrasound is the main modality of choice in the diagnosis of RPOC, the false-positive rate is 17-51% (Guarino et al., 2015). As mentioned before, on gray scale ultrasound a solid, echogenic intracavitary mass that extends to the endometrium can be seen. There is a high vascularity seen on color Doppler and a high-velocity, low-resistance flow seen with the spectral Doppler. The diagnosis of RPOC can be difficult as the ultrasonic features described above can also be found in a normal postpartum involution of the uterus, necrotic deciduans, blood clots and other diseases. CT and MRI can help with further information, detection, localisation and characterization in some difficult cases but are never used as a first line imaging method (Lee et al., 2010). The CT scan is good for a more precise anatomic location or to show a large arterial haemorrhage as a site of the extravasation of intravenous contrast material. In case of RPOC you can see an enhancing, heterogeneous mass inside the cavity. For the detection of RPOC, MRI is more useful than CT because there is more soft tissue detail. MRI should only be used if the US findings are inconclusive and if the treatment depends on further imaging. On MRI, RPOC are seen as a soft-tissue mass inside the uterus cavity with variable degrees of enhancing tissue and myometrial thinning, and obliteration of the junctional zone. A failed pregnancy, RPOC, gestational trophoblastic disease (GTD), a mole and arteriovenous malformations (AVM) could look the same with these imaging techniques. In the first trimester it is essential to differentiate between AVM, RPOC and GTD. The serum level β-human chorionic gonadotropin can be a helpful factor to distinguish between the previous mentioned conditions. While the level of β-human chorionic gonadotropin is normal or mildly elevated with RPOC, it is negative in case of AVM and elevated with GTD.

Another technique that can be used in the diagnosis of RPOC is sonohysterography. In the prospective study of Cosmi et al. (2010) they compared the accuracy of trans-vaginal ultrasound and SHG. Eighty-four patients with PPH who were suspected to have RPOC regarding their clinical signs and symptoms all underwent trans-vaginal US and SHG. The team also performed a uterine cavity curettage in all women and sent the material to the pathologic examination. Sixty out of these 84 patients were revealed to have residual trophoblastic tissue on the trans-vaginal ultrasound. With SHG they saw RPOC in 48 out of 84 patients and blood clots in 12 cases. The pathological examination showed exactly the same results as the SHG did: RPOC in 48 women and blood cloths in 12. They concluded that SHG shows a greater accuracy than trans-vaginal ultrasound in the detection of RPOC, but 15 women (17,9%) showed complications after SHG. Thirteen patients (15,5%) had a postprocedural fever and 2 women (2,4%) even had serious complications of infection. One of them had salpingitis and pelvic abscesses and needed surgery and the other one needed to be hospitalized for 10 days for fever and mild abdominal pain. Cosmi et al concluded that the adverse effects of SHG do not compensate the extra value SHG brings to make the diagnosis of RPOC and thus SHG is not recommended to perform.

In Table III there is ‘summary data of other imaging techniques’ to be found.

**Table III:** Summary data of other imaging techniques.

Imaging technique	Characteristics RPOC	Diagnostic value in comparison to gray scale US	Comments
3D Ultrasound	Large uterine cavity	No or little extra value	
Color Doppler	Hypervascularity in thickened EEC of intra- cavitary mass	Inconclusive. Increase PPV° / no increase in PPV	Absence of hypervascularity does not ex- clude RPOC
CT	Enhancing, heterogenous mass inside cavity	More precise anatomic location	Never first line investigation
MRI	Soft-tissue mass inside cavity	More detail; used if US findings are inconclusive and treatment depends on imaging	Never first line investigation
SHG		Greater accuracy than trans- vaginal US	Not recommended due to complications: fever, infection, hospitalisation

° PPV = Positive Predictive Value

### Differentiation between RPOC and arteriovenous malformation

Another less common cause of PPH is an acquired uterine arteriovenous malformation (AVM). The true incidence is not known but it is most likely over-diagnosed because of its resemblance with the far more common retained products of conception (Sellmyer et al., 2013). Most of the times AVM is acquired due to instrumentation of the uterus, the congenital type is far less frequent. The main symptoms are intermittent, disproportionate postpartum vaginal bleeding, pelvic pain, dyspareunia and sometimes high-output cardiac failure (Laifer-Narin et al., 2014). Doppler ultrasound is the golden standard diagnostic tool for AVM. Like RPOC, an echogenic mass within the endometrium can be seen but when there are also hypoechoic areas within the myometrium with high velocity and/or multidirectional vascular flow, the scan is very suggestive for AVM. If there’s a vascular component seen in RPOC, it will be located in the endometrium, whereas the vascular component in AVM is primarily situated in the myometrium (Sellmyer et al., 2013). Thereafter the patient should undergo a CT angiography and finally arteriography for confirmation of the diagnosis and possible embolization (Scribner and Fraser, 2016).

## Discussion

Retained products of conception (RPOC) are the persistency of intrauterine tissue, developed during pregnancy, after delivery. It is a serious condition and patients most frequently present with abdominal pain, fever and postpartum hemorrhage (Adkins et al., 2016). However, a diagnosis is not easy to make and ultrasound images are often used. To differentiate a normal postpartum uterus and an abnormal one, it is important to understand how a normal one is identified on ultrasound. Sokol et al. (2004) concluded that a thickened endometrial stripe and echogenic material in the cavity can indicate a normal postpartum uterus and should not be considered abnormal in every case.

In the diagnosis of RPOC the radiologist or gynecologist should be focused on a thickened endometrial echo complex (EEC) on ultrasound image, with a cut-off value of 10 mm (range between 8 and 13 mm) or an intracavitary mass, with a thickened EEC being the most sensitive finding on US correlating with RPOC or the most accurate individual predictor ( Pather et al., 2005; Sellmyer et al., 2013). The specificity on the other hand is rather low. If there is no endometrial mass found or the endometrium thickness is less than 10 mm, RPOC are rare. Other studies confirm these statements.

The combination of clinical symptoms and sonographic findings improve the diagnostic accuracy (Neill et al., 2002). Hoveyda & MacKenzie (2001) on the other hand did a study about the same topic, but concluded different findings. They stated that ultrasound imaging does not provide a more accurate diagnosis of RPOC in comparison with a clinical evaluation alone.

As indicated in most of the studies or researches consulted in this review, the use of gray scale ultrasound is important in the diagnosis of RPOC. However, there is still no consensus on a golden standard for the diagnosis of RPOC and other imaging techniques are available for diagnosis. First, the value of the 3D ultrasound is tested in the study of Belachew et al. (2015). They came to the conclusion that the 3D image does not add extra diagnostic value compared to 2D. Secondly, Urner et al. (2014), Sellmyer et al. (2013), Mulic- Lutvica et al. ( 2009), Matijevic et al. (2009), Van den Bosch et al. (2002), Steinkeler et al. (2012) and Kamaya et al. (2009) all researched the use of color Doppler ultrasound to visualize the vascularization of the echogenic mass found with gray scale US. The detection of hypervascularity in a thickened EEC or intracavitary mass is very sensitive for RPOC and the PPV increases to 96%. Although Pather et al. (2005) did not agree with this statement and they concluded that the PPV does not increase with color Doppler. However, even if there is no hypervascularity in the intracavitary mass or thickened EEC, this does not exclude RPOC. Hypervascularity is more common in RPOC, but is not a necessity (Sellmyer et al., 2013). The research of Mulic-Lutvica et al. (2009) concluded that the use of color Doppler has limited powers in the diagnosis of RPOC and the specificity of hypervascular areas is also low. Third, a CT or MRI image can be used, but never as first line techniques. Last, Cosmi et al (Cosmi et al., 2010) researched the value of sonohysterography (SHG) in the diagnosis of RPOC. They concluded that SHG is more accurate to diagnose RPOC, but the adverse effects women get due to this procedure such as fever and infections, do not compensate for the extra diagnostic value it brings.

The biggest strength of this review is the broad search for articles regarding the topic of RPOC; a limitation on the other hand is the fact that we only used English articles in the review. Articles in other languages could be beneficial as well, but we did not consult these.

## Conclusion

Momentarily there is no standardised procedure or protocol neither to perform nor to interpret postpartum ultrasound and even though more and more studies are being done, the results contradict each other. In suspicion of RPOC, the radiologist or gynaecologist should look for a thickened EEC or intracavitary mass when suspecting RPOC and a color Doppler US can be used to visualize the hypervascularisation of RPOC. However, all these findings are neither conclusive nor specific, and do not need to be present in every case of RPOC. More research needs to be done on the comparison of normal and pathological findings with trans-abdominal and/ or trans-vaginal ultrasound in order to generate a protocol to make a quick diagnosis of RPOC.

## Conflict of interest

The auteurs confirm that there is no conflict of interests regarding this literature study.
